# The Nation's Million for Nurses

**Published:** 1918-11-16

**Authors:** 


### THE MATRONS' AND SISTERS' DEPARTMENT.

### THE NATION'S MILLION FOR NURSES.

### "Render unto Cæsar the Things that are Cæsar's."

The fact that Ireland has succeeded in raising £10,000 in a few months for the benefit of the trained nurse is an object lesson to Great Britain, and offers food for serious reflection. Ireland is a country with a small population, much smaller than that of London, and the giving power of the country, based on Inland Revenue statistics of wealth, is about one twenty-fifth of that of England and Scotland. It follows that if the Irish tribute amounts to £10,000 we should have already collected in Great Britain a quarter of a million.

Now the promoters of the Irish Tribute, according to an Irish correspondent, held the view that if they waited until the end of the war before launching their appeal, their chances of success would be small, and, whether this is a correct conclusion or not, it is manifest that they made no mistake in selecting what appeared to them to be the psychological moment, and we have much pleasure in offering our congratulations. So far as Great Britain is concerned, the war being at an end, and the Tribute only in its initial stage, there is no alternative choice. Ours must of necessity be a post-war enterprise. This being so, there is no doubt that the nation's debt to the nurse must be submitted without delay. It ought to be easily possible to collect in Great Britain a Nurse's Million, always provided that the task is undertaken with zest, and while the heroism, the devotion, and the actual worth of the nurse's services in the war are fresh in the public memory. To delay further will, obviously, be fatal. It is not a charity, but a debt, and the bill must be presented forthwith. The moment hostilities are ended the process of demobilisation will begin, and the nurse, as well as the soldier, will begin to return to the Homeland. There is just a danger that the claims of the nurse may be forgotten, and we, therefore, desire to sound a note of warning. When Florence Nightingale came home from the Crimea, crowned with laurels, a grateful nation expressed its approval of her work by presenting her with a cheque for £50,000. That is sixty-four years ago or thereabouts, when the purchasing power of a sovereign was quite ten times what it is to-day. Moreover, the Crimean campaign was a military side-show in comparison with the world war in which this generation has been engaged.

Every British household owes a debt to the British nurse. Without her help many of the men who have suffered wounds, and who are now on their way to health, would have been, if not dead, permanent human wrecks. Medical and surgical skill have played an immensely important part in the programme. But without the constant devotion and skill of the nurses much of their labour would have been in vain. These women have, since August 1914, carried themselves with a spirit of courage and patriotism worthy of the highest traditions of the race. The bombing of Red Cross hospitals and the sinking of Red Cross ships have never deterred them from doing their duty. In the deep sea the bones of British nurses lie, and the "Last Post" has sounded over their graves at the battle front. There have been no hysterics, no cowardice, no flinching. To the fullest they have responded to the call, and wounded Tommy and wounded Officer will alike testify to the fact that they owe much to the skill and devotion of the trained nurse, the great sisterhood of mercy that Florence Nightingale bequeathed, not merely to the nation, but to the world.

Now is the moment for action, for vigorous and whole-souled propaganda. The nurse is the child of war. It is her proud privilege to carry the cross of healing by sea and land wherever British soldiers fight. Husband and father, wife and mother, owe a tribute to the nurse. The hour strikes when this tribute has to be paid. Let us call it the Nurses' Million, and let it be gathered in while the song of victory is being sung. Never again must the British nurse be consigned, when her work is done, to the almshouse or the workhouse. Her rightful place is amongst the nation's heroes. But, if she is to enjoy the fruits of her labour, her cause must be championed, and without delay. No stone must be left unturned to ensure that the nurse's claim on the nation is promptly submitted. If this be done, we believe it will be met in a spirit of generosity and righteousness.

